# Cross-National Systematic Review of Neonatal Mortality and Postnatal Newborn Care: Special Focus on Pakistan

**DOI:** 10.3390/ijerph14121442

**Published:** 2017-11-23

**Authors:** Mansoor Ahmed, Youngjoon Won

**Affiliations:** 1 Department of Global Health and Development & Department of Preventive Medicine, College of Medicine, Hanyang University, Seoul 04763, Korea; mansoormahar@hanyang.ac.kr; 2 Department of Information Systems, Hanyang University, Seoul 04763, Korea

**Keywords:** global health, neonatal mortality, newborn care, epidemiology, low- and middle-income countries, vulnerable populations, Pakistan, health policy

## Abstract

The latest nationwide survey of Pakistan showed that considerable progress has been made toward reducing all child mortality indicators except neonatal mortality. The aim of this study is to compare Pakistan’s under-five mortality, neonatal mortality, and postnatal newborn care rates with those of other countries. Neonatal mortality rates and postnatal newborn care rates from the Demographic and Health Surveys (DHSs) of nine low- and middle-income countries (LMIC) from Asia and Africa were analyzed. Pakistan’s maternal, newborn, and child health (MNCH) policies and programs, which have been implemented in the country since 1990, were also analyzed. The results highlighted that postnatal newborn care in Pakistan was higher compared with the rest of countries, yet its neonatal mortality remained the worst. In Zimbabwe, both mortality rates have been increasing, whereas the neonatal mortality rates in Nepal and Afghanistan remained unchanged. An analysis of Pakistan’s MNCH programs showed that there is no nationwide policy on neonatal health. There were only a few programs concerning the health of newborns, and those were limited in scale. Pakistan’s example shows that increased coverage of neonatal care without ensuring quality is unlikely to improve neonatal survival rates. It is suggested that Pakistan needs a comprehensive policy on neonatal health similar to other countries, and its effective programs need to be scaled up, in order to obtain better neonatal health outcomes.

## 1. Introduction

The target of the fourth Millennium Development Goal (MDG4) was to reduce the global number of children dying under the age of five by two-thirds between 1990–2015 [1]. However, the under-five deaths only halved to 43 deaths per 1000 live births, according to the MDG Report 2015. Nearly 80% of these deaths occurred in sub-Saharan Africa and South Asia [2]. According to the Global Health Observatory of the World Health Organization (WHO), children within the neonatal period (birth to the first 28 days) remain at the highest risk of dying, as this period comprised about 45% of all under-five deaths in 2013 [3]. Progress on reducing neonatal mortality is slower than that of under-five and infant mortality; the global neonatal mortality rate decreased from 33 per 1000 live births in 1990 to 21 in 2012 [2]. It is estimated that 99% of newborn deaths take place in low- and middle-income countries (LMICs) [4].

**Figure 1:**
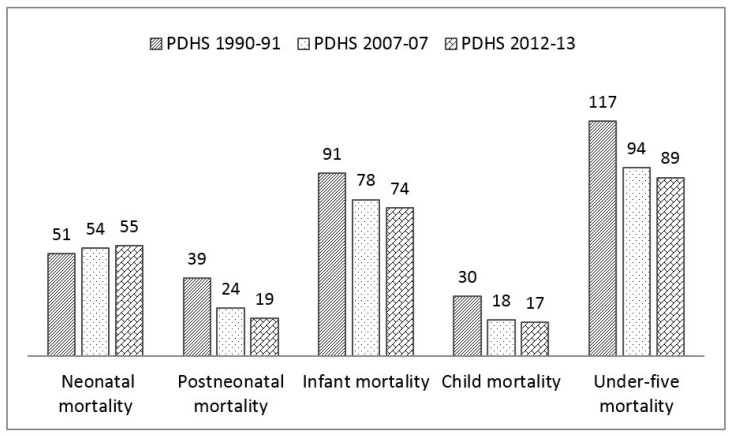
Child mortality (per 1000 live births) from the Pakistan Demographic and Health Surveys (PDHS) of 1990–1991, 2006–2007, and 2012–2013.

Even among the LMICs, there are obvious disparities in terms of the progress made towards MDG4. Such disparities could be related to socio-economic factors, health care services, and maternal, newborn, and child health (MNCH) policies [2,5]. Several countries, such as Bangladesh, Nepal, Ethiopia, and Tanzania, have already achieved the MDG4 target. However, countries such as Pakistan still lag behind. Although Pakistan is off track for achieving the MDG4 target, the Pakistan Demographic and Health Survey (PDHS) of 2012–2013 reported that considerable progress has been made in reducing all child mortality indicators except neonatal mortality [6,7] since its first survey in 1990–1991 (Figure 1). In fact, Pakistan’s neonatal mortality rate has gradually increased since 1990.

Few attempts have been made to investigate the factors associated with the increasing neonatal mortality rate in Pakistan. One study highlighted substandard maternal and newborn care and the late initiation of breastfeeding practice as possible causes [8]. Another study [9] underscored the determinants of neonatal mortality via secondary analysis of the PDHS of 2006–2007. It also demonstrated that income, male gender, first-rank baby, and below average birth size were associated with neonatal deaths. However, the following two questions still remain unanswered: (1) Are there any MNCH policies in Pakistan that emphasize newborn care? (2) If there is adequate newborn care coverage, is the outcome satisfactory as compared with other LMICs? To the best of our knowledge, very little has been done to compare neonatal mortality rates and postnatal newborn care in Pakistan with other LMICs. Cross-national research can provide useful findings based on certain performance indicators [10].

This study has two primary objectives. The first is to compare Pakistan’s progress with the selected LMICs for the two trend categories: under-five mortality to neonatal mortality, and neonatal mortality to postnatal newborn care. The second objective is to conduct an analysis of Pakistan’s MNCH policies and programs, which have been implemented since the benchmark year of 1990, to determine whether postnatal newborn care was included in the policies and programs.

## 2. Materials and Methods

**Figure 2:**
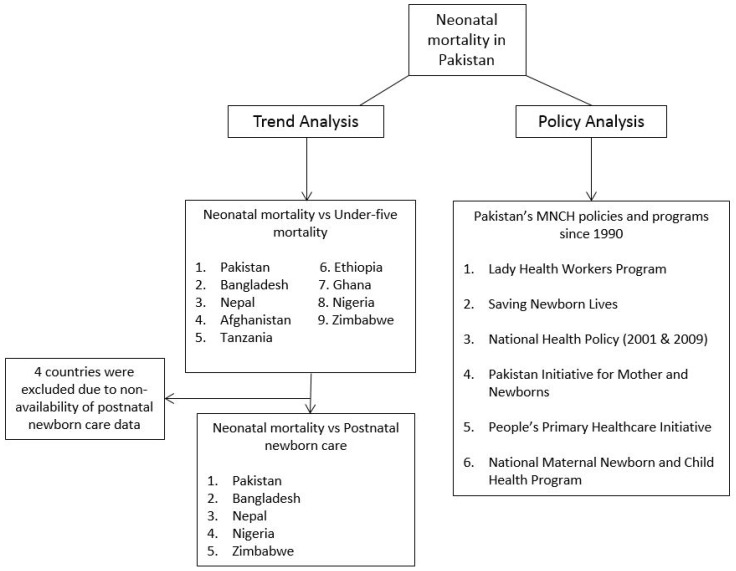
Conceptual framework illustrating trend and policy analyses for neonatal mortality in Pakistan.

To elucidate the concept of this study, a conceptual framework was designed, which is illustrated in Figure 2.

### 2.1. Data Source for Trend Analysis

Trend analysis data were obtained from 27 Demographic and Health Surveys (DHS) from nine LMIC countries, with three DHSs per country. These DHSs are publicly available on www.dhsprogram.com. A mix of Asian and African countries was selected: Pakistan, Afghanistan, Bangladesh, and Nepal from Asia; Ethiopia, Ghana, Nigeria, Tanzania, and Zimbabwe from Africa. Asian countries were arbitrarily selected because of their regional and cultural proximity to Pakistan. India would have also been selected, but there have not been DHSs in India since 2005–2006. The five African countries were randomly selected by drawing lots. In order to minimize the length time bias, only three DHSs were chosen from each country, which surveyed the years closest to Pakistan’s three DHSs.

### 2.2. Measures

The neonatal mortality rates of all nine LMIC countries were plotted against their under-five mortality rates for each country’s three DHSs. Neonatal mortality is defined as the probability of dying within the first month of life, and under-five mortality is defined as the probability of dying between birth and the fifth birthday. Both mortality rates are expressed as deaths per 1000 live births.

In this study, the relationship between the babies receiving postnatal care and neonatal mortality was observed. Although the WHO recommends at least three routine checkups for newborns in first seven days of life [11], most neonatal deaths occur in first 48 h of life, and the most significant reduction in neonatal mortality is achieved when the baby is assessed within this period [2,12]. For this study, the postnatal care for newborns is defined and presented as the “proportion of the newborns who received their first routine checkup within 48 h of life, out of the total number of live births recorded in the respective DHS’. This routine assessment is checked for danger signs, such as: no spontaneous movement, fever, low body temperature, fast breathing, convulsions, severe chest in-drawing, stopped feeding, jaundice, etc. [11]. The postnatal care measure was included in the DHS program since 2010 [6]. Thus, only five countries were eligible for the selection: Bangladesh, Nepal, Pakistan, Nigeria, and Zimbabwe.

### 2.3. Demographic and Health Surveys (DHSs)

The DHS program is jointly funded by the United States Agency for International Development (USAID), international donor agencies, and the host country. Since 1984, it has collected, analyzed, and disseminated nationally representative data on health, family planning, and nutrition via over 300 household surveys in more than 90 developing countries. Each DHS provides a vast repository of information on key health indicators. Its main purpose is to collect data that can be easily compared across different countries. For comparison across countries, standard model questionnaires are developed and revised over the six phases of the DHS to suit the requirements of each country. Ideally, a country should fully adopt the model questionnaire, but can add or delete the questions if needed.

For the countries included in this study, there could be some variation between sample sizes and the survey instruments used for data collection. Typically in a DHS, a multi-stage stratified sample design is adopted. The details of the data collection process are available in the final reports, which can be freely accessed from www.dhsprogram.com. Table 1 presents the data collection period, the total number of households, the total number of women interviewed for child mortality, and the total number of births, in order to calculate postnatal newborn care according to each country. Details on the data collection process for the Pakistan DHS of 2012–2013 are given here.

### 2.4. Pakistan DHS of 2012–2013

The Pakistan DHS of 2012–2013 was conducted from October 2012 to March 2013. A two-stage stratified sampling method was adopted so that the generated sample could be representative of the country. These samples from urban and rural areas were drawn separately from six administrative units of Pakistan, as defined by the 1998 population census: four provinces, Gilgit-Baltistan, and the Islamabad Capital Territory. A total of 500 primary sampling units (PSUs) were selected in the first stage using a probability proportional to the size and sampling scheme. The PSUs consisted of 252 rural areas and 248 urban areas. A fixed number of 28 households per each PSU were selected in the second stage using a systematic sampling technique with a random start. A total of 12,943 households were interviewed, where 6608 were in rural areas and 6335 were in urban areas.

The child mortality and postnatal newborn care rates were calculated using information obtained from 13,558 ever-married women aged 15 to 49 years. They were asked to report the death of any child born in the five years preceding the survey, and the age at death was recorded in days for those dying in the first month of life. The postnatal newborn care was recorded for the last-born child to an eligible woman for the two years preceding the survey.

### 2.5. Data Source for Policy Analysis

This study analyzed Pakistan’s MNCH policies and programs, which have been implemented since the benchmark year of 1990 (the year of the first PDHS). Policy review is reported in accordance with the PRISMA guidelines (preferred reporting items for systematic reviews and meta-analyses) [13]. We performed a search on Google Scholar and the PakMediNet database for the relevant policies and programs conducted between 1990 and July 2017. The search was conducted using Boolean operators, and different keywords were used alone and in combinations. The full electronic search strategy for Google Scholar is provided as supplementary material in Table S1. The bibliographies of the relevant materials were also searched. The identified documents were then screened through the following inclusion criteria:

1. any health policies or programs related to maternal, newborn, and child health that started after the year 1990, and are still in progress;
2. public, private, semi-private, and non-governmental organization programs;
3. implemented in more than 20 districts.

These criteria yielded two health policies and five other programs related to MNCH in Pakistan. These programs were thoroughly read by the two authors, and relevant data were extracted, such as the: title of the program/policy, year started, scale of the program (whether nationwide or limited to certain regions), goals and objectives of the program/policy, and MNCH components.

### 2.6. Data Analysis

Information on MDG4 progress (the expected year of achieving the MDG4 target) was retrieved from mdgTrack (www.mdgtrack.org), based on the “MDG progress classification” by the United Nations Economic and Social Commission for Asia and the Pacific in 2010 [14]. OriginPro 9.0.0 software (OriginLab Corporation, Northampton, MA, USA) was used to illustrate the disparities among countries in the trend analysis that is presented in the table and figures.

## 3. Results and Discussion

To the best of our knowledge, this is the first cross-national comparative study that focuses on the neonatal mortality and postnatal newborn care rates of LMICs. The most significant findings are: (1) in spite of having the highest rate of postnatal newborn care among the selected countries, Pakistan’s neonatal mortality rate is the highest; (2) its neonatal mortality rate also accounts for the highest proportion of under-five deaths; (3) Pakistan is the only country where under-five mortality is decreasing and neonatal mortality is increasing; and (4) the country has no nationwide policy with a special focus on neonatal health.

### 3.1. Neonatal Mortality Rate

**Figure 3:**
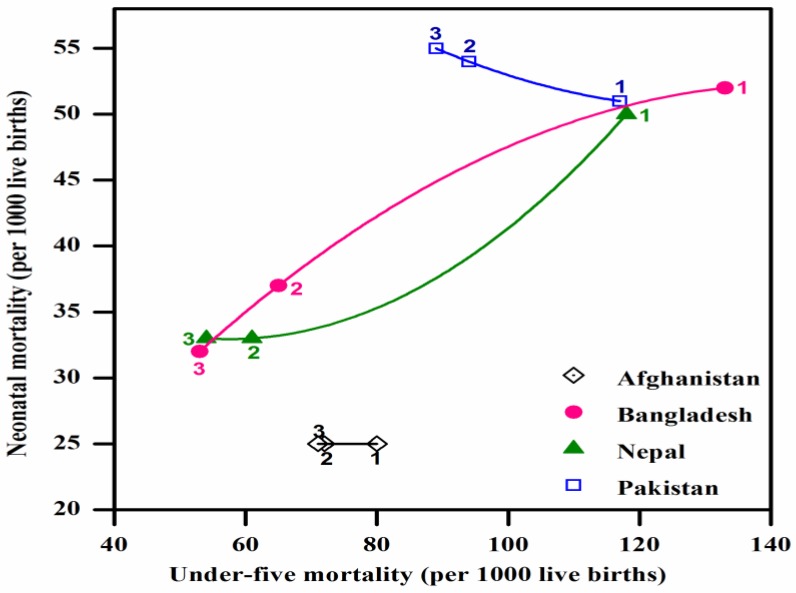
Under-five mortality vs. neonatal mortality: Pakistan and other Asian countries. Each data point is labelled 1, 2, and 3 for each country, where 1 and 3 denote the earliest and latest DHS, respectively.

**Figure 4:**
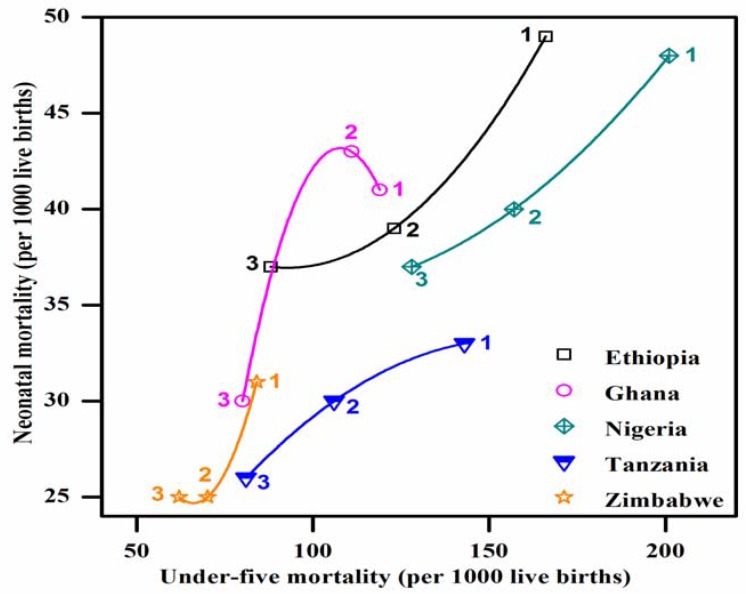
Under-five mortality vs. neonatal mortality: African countries. Each data point is labelled 1, 2, and 3 for each country, where 1 and 3 denote the earliest and latest DHS, respectively.

**Figure 5:**
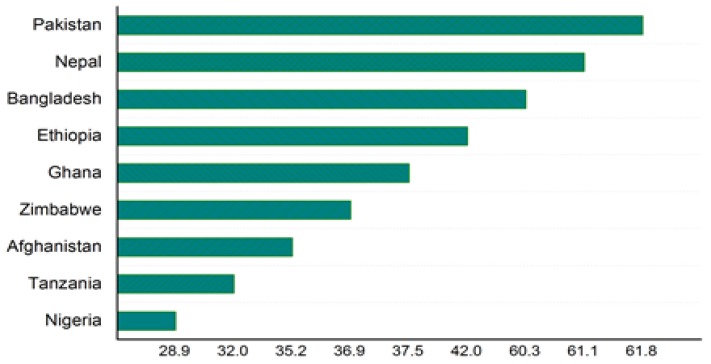
Neonatal mortality as proportion of the under-five mortality rate, according to the latest DHS.

Table 2 presents the list of examined countries and their trends of their under-five to neonatal mortality rates since 1990. Figure 3 and Figure 4 illustrate the trends of the under-five and neonatal mortality rates in Asian and African countries. Pakistan’s neonatal mortality rate is inversely proportional to its under-five child deaths. Its under-five mortality has decreased from 117 in 1991 to 89 in 2012–2013; whereas the neonatal mortality rate has slightly increased, from 51 to 55 over the same period. In Zimbabwe, both mortality rates have increased over 15 years. The under-five mortality rate in Bangladesh has decreased from 133 in 1992 to 53 in 2011, and its neonatal mortality has decreased from 52 to 32. The neonatal mortality rates in Nepal and Afghanistan remain unchanged for the previous two surveys. The rest of the countries have shown a continuous decreasing trend. These results demonstrate that among the countries included in this study, Pakistan shows abnormal behavior, with an increase in only the neonatal mortality rate. Table 2 shows each country’s MDG4 status. Nepal (2010), Bangladesh (2012), Tanzania (2013) and Ethiopia (2014) have already achieved MDG4. Figure 5 presents the neonatal mortality rates as a percentage of the under-five mortality rate among the countries included in this study, according to each country’s latest DHS. Pakistan’s neonatal mortality rate has the highest proportion (61.8%) within the under-five mortality rate, followed by Nepal (61.1%), Bangladesh (60.3%), Ethiopia (42.0%), and Ghana (37.5%).

Zimbabwe presents a very unusual case here; its under-five and neonatal mortality rates have increased from 62 to 84 and 25 to 31 per 1000 live births (1999–2011). These figures could be related to the country’s economic meltdown in those years, which was worst in 2008. During this period, the life expectancy in Zimbabwe declined to 34 years for women and 37 years for men [15], which was the lowest in the region. In Ghana, the government has adopted The Ghana National Newborn Health Strategy and Action Plan 2014–2018 [16]. Bangladesh introduced a National Neonatal Health Strategy and Guidelines in 2009 to achieve sustainable progress towards MDG4 [17]. In the following year, Bangladesh was rewarded with a United Nations award for the acknowledgement of its steady progress in reducing neonatal and maternal mortality rates [18]. Moreover, several other initiatives such as Saving Newborn Lives (SNL) played a key role in increasing the number of newborns receiving postnatal care, and subsequently, Bangladesh achieved MDG4 in 2012 [14,19]. Similarly, Nepal achieved its targets for MDG4 in 2010. Its accomplishment was attributed to the community-based approach to health care by moving the essential MNCH services nearer to the most vulnerable populations [20]. Nepal adopted its National Neonatal Health Strategy in 2004, which was further bolstered by the Community-Based Newborn Care Program from 2007; these programs significantly contributed toward improving the health and survival of newborns [21]. Details on Pakistan’s MNCH policies and programs are discussed later in our policy analysis.

Decreases in neonatal mortality, when isolated from under-five mortality and compared with the changes in under-five mortality, are consistently slower in several countries, including those reviewed in this study. The sluggish progress on reducing neonatal mortality could be due to the lack of continuity in care from delivery to newborn care [22]. Since 1990, significant progress has been made toward achieving the MDG4 target, but little attention has been paid to neonatal health [5]. In fact, neonatal mortality is not specified as an MDG4 indicator, unlike under-five and infant mortality. Consequently, the child health programs have specifically targeted children over one month of age, and the postnatal care programs have neglected newborn care [23]. Following the WHO’s recommendations, several countries have recently been incorporating neonatal health into their postnatal programs [24]. As a result, the latest DHS includes postnatal newborn care as one of the performance indicators [6,25,26,27,28].

### 3.2. Ineffective Postnatal Care

**Figure 6:**
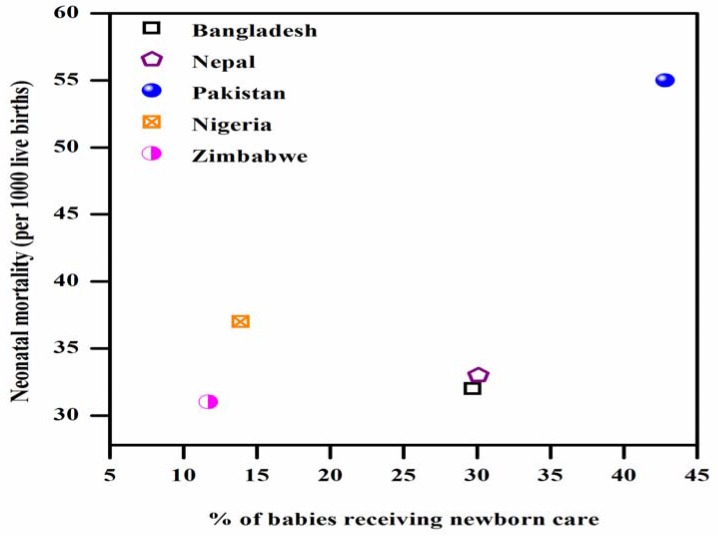
Relationship between neonatal mortality and postnatal care within the first 48 h of life.

Figure 6 presents the proportion of babies receiving newborn care with respect to neonatal mortality. The neonatal mortality rate in Pakistan is highest (55 per 1000 live births) among the countries reviewed in this study, and its proportion (42.8%) of first postnatal newborn care within 48 h of life is also the highest. Nigeria’s neonatal mortality is 37 (per 1000 live births), with 13.9% of babies receiving postnatal care. Zimbabwe shows a mortality of 31 (per 1000 live births), with 11.7% of babies receiving postnatal care. Among the other studied Asian countries, Bangladesh’s neonatal mortality is 32 (per 1000 live births), with 29.7% of babies receiving postnatal care, and Nepal shows neonatal mortality of 33 (per 1000 live births), with 30.1% of babies receiving postnatal care.

Pakistan’s poor progress on reducing neonatal mortality has been previously reported [7,8,18,19]. However, this study demonstrates for the first time its possible relationship with postnatal newborn care. Previously, a cohort study from Pakistan’s urban population, which has access to skilled care, had highlighted quality issues in newborn care [29]. It concluded that the quality of postnatal care was unsatisfactory, despite good access to newborn care. Elsewhere, a study from Ghana identified that the quality of care in the neonatal department was the lowest among all of the departments in the hospitals [30]. In this study, Pakistan’s case shows that increased health care coverage, without improving the quality in care, is unlikely to have a significant impact on neonatal survival. The authors recommend the adoption of the WHO Safe Childbirth Checklist program to overcome the quality issues in MNCH care services [31]. Recently, a pilot study reported significant improvement in the quality of maternal and perinatal health outcomes following the implementation of this program [32].

The findings also highlighted that Bangladesh, Nepal, and Pakistan (Asian countries) have 30% or higher coverage for postnatal newborn care. Conversely, Nigeria and Zimbabwe (African countries) have 15% or lower coverage. One possible explanation for such disparity between Asian and African countries could be that most of the African countries have been more attentive toward HIV/AIDS and malaria, and newborn health received inadequate attention as a consequence. Thus, there is a need to develop capacity, ensure sustainable support, and work with government agencies to incorporate newborn care into the health programs [33].

### 3.3. No Nationwide Policy on Newborn Care

**Figure 7:**
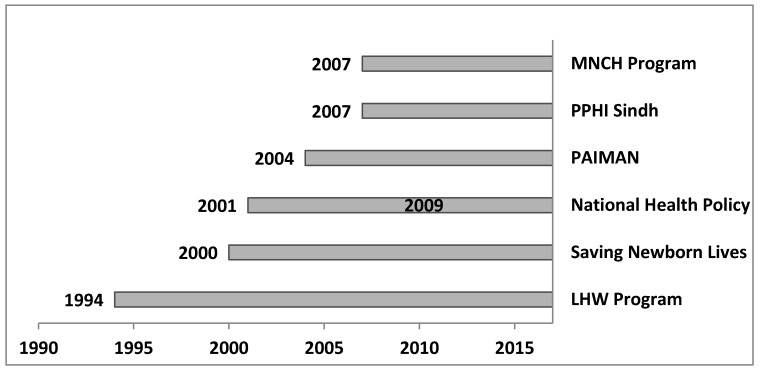
Timeline showing the starting year of health policies and programs with maternal, newborn, and child health (MNCH) components in Pakistan.

Figure 7 illustrates the timeline of Pakistan’s MNCH policies and programs implemented since 1990. Over this period, two national health policies and five other programs with MNCH components, were introduced. Table 3 presents their starting year, scale of coverage, and their summary, with a special focus on MNCH components. The policy analysis shows that no comprehensive nationwide policy focusing on neonatal health exists in the country. Out of the seven programs, only two focused on neonatal health: Saving Newborn Lives (SNL) and the Pakistan Initiative for Mothers and Newborns (PAIMAN). However, both programs are limited to only few districts.

SNL is executed by an international non-governmental organization (Save the Children) with the support of foreign donors; the program supports newborn care through the effective mobilization of Lady Health Workers (LHWs) and traditional birth attendants (TBAs) [35]. Its intervention is limited to only a few districts. In those districts, the skilled deliveries increased up to 30% and newborn mortality fell by 28% [35]. The PAIMAN is mainly supported by the USAID. Its primary objective is to upgrade existing public health programs [8]. It has achieved significant results, such as a decrease in neonatal mortality rates and increase in the proportion of skilled deliveries; however, similar to SNL, PAIMAN’s coverage has also been limited to only a few districts [37]. LHWs are the only health worker cadre that deliver essential services to the rural population in Pakistan [41]. However, the Lady Health Workers Program (LHWP) is limited in its scope to educate and counsel on family planning and institutional delivery, promote appropriate feeding practices, and carry out routine and polio immunization activities [42,43]. The Maternal, Newborn and Child Health (MNCH) program is a nationwide landmark program, because in order to achieve the targets of MDG4 and 5, it introduced a new a cadre of community-based skilled birth attendants called community midwives [44]. The midterm evaluation of the program showed that it could not be translated into an effective MNCH program, and was likely to be unsustainable, because the relevance of the program had been eroded by poor planning, a lack of leadership, and poor management [38]. The People’s Primary Health Care Initiative (PPHI) was started as a pilot program in Sindh province though a public–private partnership [45]. The primary objective of this program was to guide the efficient service delivery of first-level health facilities such as basic health units (BHUs), dispensaries, and Mother and Child Health Centers (MCHCs) through mobilizing human resources, developing physical infrastructure, making MCHCs operational, encouraging community participation, and several other initiatives [46]. The National Health Policy of 2009 endorsed the execution of the MNCH components of existing programs such as the Expanded Program on Immunization (EPI) and the MNCH program, and promised to ensure round-the-clock delivery of quality maternal care [40]. However, progress toward this objective has been very slow [41].

The implementation of neonatal programs using effective interventions is crucial for decreasing neonatal mortality rates in developing countries [47]. Additionally, we recommend that under the umbrella of sustainable development goals, countries should include separate measures for tracking neonatal health and mortality, so that future programs and international supports are more dedicated to this issue. This is becoming increasingly necessary, as progress on reducing neonatal mortality rates has been consistently slower in several countries, as discussed earlier. There is a need to create a healthy environment for children, especially those from vulnerable populations such as LMICs. The authors recommend the introduction of a nationwide policy for neonatal health in Pakistan, which should prioritize high-quality postnatal care for newborns through introducing cost-effective, community-based approaches. Along with the provision of routine care, there is need for the specialist care for small preterm newborns and babies with special conditions such as neonatal encephalopathy. Moreover, as part of the policy for neonatal health, coverage of already proven existing programs such as SNL and PAIMAN should be scaled up. The main objective should be to end preventable newborn deaths, as emphasized by the UN’s Every Newborn Action Plan [48].

### 3.4. Limitations

The trend analysis is based on the nationally representative data of nine countries, which is the biggest strength of this study. It has a few limitations that need to be considered. First, the DHSs for all of the countries reviewed in this study were not conducted over the same period, and there was a significant difference in sample sizes; thus, it might have affected the estimations and comparisons of newborn care and mortality. Nevertheless, in the DHSs, the same methodology and questionnaires were used to measure health indicators across different sites, allowing comparability across populations [49]. Therefore, the results present a reasonable estimate of each country’s progress on neonatal mortality as compared with other LMIC countries. Second, due to unavailability of the data, postnatal newborn care for all nine countries could not be presented. Moreover, it was not possible to estimate how much neonatal mortality could be prevented by 100% postnatal care coverage with the data in hand. Third, there is a possibility of recall bias, because the participants were required to report events over a five-year period. Another possibility is that the mothers might have been hesitant to discuss matters associated with the death of their children, which would have resulted in the underreporting of mortality rates. Fourth, neonatal mortality is a component of under-five mortality, so minor changes in the proportion of rates may be influenced by small changes in the estimated age of death. Furthermore, the countries for DHS analysis were selected arbitrarily and randomly, rather than systematically.

## 4. Conclusions

This is the first cross-national study to emphasize the quality of neonatal health care. Our findings suggest that there is a need to implement a continuum of care approach from the delivery of the child through its first month of life through effective postnatal newborn care. In Pakistan, there is no nationwide policy on newborn health, and programs focusing on newborn care are limited in coverage. Therefore, the proven and cost-effective programs need to be scaled up in order to achieve significant improvements in neonatal health outcomes.

## Figures and Tables

**Table 1:** Characteristics of the Demographic and Health Survey (DHS) according to country.

Data Point	Country	Survey Period	Total No. of Households Interviewed in the DHS	No. of Women Interviewed for Child Mortality	Total No. of Births for Postnatal Newborn Care
1	Pakistan	1990–1991	3591	3227	-
	Afghanistan	1995–1996	-	-	-
	Bangladesh	1993–1994	9174	8174	-
	Nepal	1996	8082	8429	-
	Zimbabwe	1994	5984	6128	-
	Ghana	1993–1994	5822	4562	-
	Nigeria	2003	7225	7620	-
	Ethiopia	2000	14,072	15,367	-
	Tanzania	1996	7969	8120	-
2	Pakistan	2006–2007	92,340	10,023	-
	Afghanistan	2006–2007	8278	8281	-
	Bangladesh	2007	10,400	10,996	-
	Nepal	2006	8707	10,793	-
	Zimbabwe	2005–2006	9285	8907	-
	Ghana	2003	6251	5691	-
	Nigeria	2008	34,070	33,385	-
	Ethiopia	2005	13,721	14,070	-
	Tanzania	2004–2005	9735	10,329	-
3	Pakistan	2012–2013	14,000	13,558	4246
	Afghanistan	2010	22,351	47,848	-
	Bangladesh	2011	17,141	17,842	4652
	Nepal	2011	10,826	12,674	2030
	Zimbabwe	2010–2011	9756	9171	2448
	Ghana	2008	11,778	4916	-
	Nigeria	2013	38,522	38,948	12,473
	Ethiopia	2010–2011	16,706	16,515	-
	Tanzania	2009–2010	9623	10,139	-

Data point: Data point 1 refers to data from the oldest DHS, and data point 3 refers to data from the latest DHS.

**Table 2:** Under-five and neonatal mortality rates from 1990 to 2013: Pakistan and eight countries from Asia and Africa tracking the fourth Millennium Development Goal (MDG4).

Countries	Data Point 1		Data Point 2		Data Point 3		Trends		Expected Year of Achieving MDG4
	Under-Five Mortality (5M)	Neonatal Mortality (NM)	5M	NM	5M	NM	5M	NM	
Pakistan	117	51	94	54	89	55	↓	↑	2025
Afghanistan	80	25	72	25	71	25	↓	no change	2025
Bangladesh	133	52	65	37	53	32	↓	↓	Achieved in 2012
Nepal	118	50	61	33	54	33	↓	no change	Achieved in 2010
Zimbabwe	62	25	70	25	84	31	↑	↑	2025
Ghana	119	41	111	43	80	30	↓	↓	2025
Nigeria	201	48	157	40	128	37	↓	↓	2025
Ethiopia	166	49	123	39	88	37	↓	↓	Achieved in 2014
Tanzania	143	33	106	30	81	26	↓	↓	Achieved in 2013

5M: Under-five mortality; NM: Neonatal mortality; ↓: decreasing trend; ↑: increasing trend; Under-five and Neonatal mortality indicators given as per 1000 live births; Data point: Data point 1 refers to data from the oldest Demographic and Health Survey (DHS), and data point 3 refers to data from the latest DHS; Expected year of achieving MDG4 from mdgtrack.org.

**Table 3:** Public health policies and programs with MNCH components in Pakistan.

Program	Year Started	Scale of the Program	MNCH Components
National Program for Family Planning and Health Care (LHW program) [34]	1994	Nationwide (rural population)	To educate all eligible couples about family planning methods and distribute contraceptives To encourage institutional delivery To provide supplements to mothers and children To promote breastfeeding and complementary feeding Immunization activities Home-based pneumonia management No technical training provided
Saving Newborn Lives [35]	2000	Few districts (rural population)	Started as a pilot in one rural district To support newborn care through lady health workers (LHWs) and traditional birth attendants (TBAs) Technical training for TBAs, but neither equipment nor injectable drugs provided Newborn mortality fell by 28% in intervention areas Skilled deliveries increased up to 30%
National Health Policy 2001 [36]	2001	Nationwide	10 key areas, two principally MNCH related: (1) Commitment to Expanded Program on Immunization (EPI) (2) To fill the nutrition gaps in children, women, and other vulnerable groups
Pakistan Initiative for Mothers and Newborns [37]	2004	24 districts	Capacity building of existing programs in targeted districts to reduce maternal, newborn, and child deaths Main outcomes were a reduction in neonatal mortality and an increase in the proportion of skilled births 97 public health facilities upgraded 2204 public health care providers trained in essential maternal and newborn care
Maternal, Newborn and Child Health Program [38]	2007	Nationwide	To reduce maternal, newborn, and child morbidity and mortality To strengthen ongoing projects and harmonize the delivery of MNCH services Health facilities at all levels to provide a comprehensive maternal and newborn health services package To ensure the delivery of quality MNCH services in 7000+ health facilities To introduce a cadre of community-based skilled birth attendants (community midwives) who would meet the international definition of skilled birth attendants To provide comprehensive family planning services at health facilities, including the provision of contraceptives
People’s Primary Health Care Initiative [39]	2007	One province (Sindh)	To guide the efficient service delivery of first-level health facilities such as basic health units (BHUs), dispensaries, and Mother and Child Health Centers (MCHCs)
National Health Policy 2009 [40]	2009	Nationwide	To expand services of nutrition, EPI, and MNCH programs To ensure the training and deployment of community midwives through the MNCH program To provide round-the-clock comprehensive and basic Emergency Obstetric and Newborn Care (EmONC) services The MNCH and LHW programs will implement the management of common childhood illnesses at facility and community levels To expand the Integrated Management of Neonatal and Childhood Illness (IMNCI) program

## Supplementary Materials

The following are available online at www.mdpi.com/1660-4601/14/12/1442/s1, Table S1: Search strategy.
