# Supplementary material for: Cross-National Systematic Review of Neonatal Mortality and Postnatal Newborn Care: Special Focus on Pakistan
Source: Int J Environ Res Public Health. 2017 Nov 23;14(12):1442. doi: 10.3390/ijerph14121442 (PMC5750861; doi:10.3390/ijerph14121442)
Supplement: Supplementary file 1 [file ijerph-14-01442-s001.pdf]

Cross-national Systematic Review on Neonatal Mortality and Postnatal Newborn care: Special focus on Pakistan

Supplementary Data (Web Only Files)

Mansoor Ahmed and Youngjoon Won*

Table S1. Google Scholar search strategy for studies to include in systematic review.

1. Pakistan
2. Healthcare OR health care
3. National OR provincial OR regional
4. Maternal and child health
5. Maternal newborn and child health
6. Health policy
7. Health program
#1 AND #2 AND #3 AND (#4 OR #5 OR #6 OR #7)
